# Blood Cell In Vitro Cytokine Production in Response to Lipopolysaccharide Stimulation in a Healthy Population: Effects of Age, Sex, and Smoking

**DOI:** 10.3390/cells11010103

**Published:** 2021-12-29

**Authors:** Lluis Rodas, Sonia Martínez, Aina Riera-Sampol, Hannah J. Moir, Pedro Tauler

**Affiliations:** 1 Research Group on Evidence, Lifestyles and Health, Department of Fundamental Biology and Health Sciences, Research Institute of Health Sciences (IUNICS), University of the Balearic Islands, 07122 Palma, Spain; lluisrodas@hotmail.com; 2 Research Group on Evidence, Lifestyles and Health, Department of Nursing and Physiotherapy, Research Institute of Health Sciences (IUNICS), University of the Balearic Islands, 07122 Palma, Spain; ana.riera@uib.es; 3 Health Research Institute of the Balearic Islands (IdISBa), 07120 Palma, Spain; 4 School of Life Sciences, Pharmacy and Chemistry, Faculty of Science Engineering and Computing, Kingston University London, Penrhyn Road, Kingston upon Thames KT1 2EE, UK; H.Moir@kingston.ac.uk

**Keywords:** LPS challenge, blood culture, age, sex, smoking, menstrual cycle

## Abstract

Immune system functionality has been commonly assessed by a whole-blood or isolated-cell stimulation assay. The aim of this study was to determine whether cytokine production in whole-blood-stimulated samples is influenced by age, sex, and smoking. A descriptive cross-sectional study in 253 healthy participants aged 18–55 years was conducted. Whole blood samples were stimulated for 24 h with LPS and concentrations of IL-6, IL-10, and TNF-α were determined in the culture media. Among parameters considered, statistical regression analysis indicated that smoking (change in R^2^ = 0.064, *p* < 0.001) and sex (change in R^2^ = 0.070, *p* < 0.001) were the main predictors for IL-10 production, with higher values for women and non-smokers. Age was also found to be a significant predictor (change in R^2^ = 0.021, *p* < 0.001), with higher values for younger ages. Age (change in R^2^ = 0.089, *p* = 0.013) and smoking (change in R^2^ = 0.037, *p* = 0.002) were found to be negative predictors for IL-6 production. Regarding TNF-α-stimulated production, age (change in R^2^ = 0.029, *p* = 0.009) and smoking (change in R^2^ = 0.022, *p* = 0.022) were found to be negative predictors. Furthermore, sex (change in R^2^ = 0.016, *p* = 0.045) was found to be a significant predictor, with lower values for women. In conclusion, sex, age, and smoking were found to be independent determinants of stimulated cytokine production. While female sex is associated with higher IL-10 and lower TNF-α production, aging and smoking are associated with lower IL-6, IL-10, and TNF-α production.

## 1. Introduction

The capacity of leukocytes to produce cytokines upon adequate challenge has been considered an interesting question with potential consequences for the entire functional capacity of the immune system [1]. In this regard, immune responsiveness has been commonly assessed by a whole-blood or isolated-cell stimulation assay, which measures the culture concentration of cytokines produced by immune cells upon stimulation with, among others, the Gram-negative stimulus lipopolysaccharide (LPS). [2]. This stimulation assay primarily assesses individuals’ cytokine production by monocytes [3], a process reported to be under genetic control [4]. Monocyte-derived cytokines are involved in immune responses such as the activation of B cells, leading to the production of specific antibodies, the activation of T cells, leading to cell-mediated immunity, and the enhancement of several neutrophil and macrophage/monocyte functions [5]. Consequently, monocyte-derived cytokines play important roles in host defense against bacterial infections [5].

Measurement of LPS-stimulated cytokine production has been used as a model of immune responsiveness in non-infectious diseases [2]. A low stimulated cytokine production has been associated with increased mortality [6], metabolic syndrome and type 2 diabetes [7], lower serum triiodothyronine levels [8], and lower muscle mass and strength [9], all of which are considered as consequences of the aging process. Furthermore, it has been reported that monocytes from depressed patients present an altered response to endotoxin stimulation [10]. However, whether the cytokine production response plays a causal role in the development of these diseases still needs to be determined [2].

The effect of sex on LPS-stimulated cytokine production remains unclear. While some studies have reported a higher stimulated cytokine production in men than in women [2,11,12,13], others have found no sex differences [14]. In this regard, age has been suggested to be an important factor, as sex differences seem to be higher in young people [2]. Regarding age, the influence of aging on the production of cytokines by monocytes has also been investigated, and an impaired ability to produce interleukin (IL)-1β and tumor necrosis factor (TNF)-α with aging has been shown [5,11,15]. However, most of these studies have compared the stimulated cytokine production in elderly (older than 70 years) and in young (around 25–30 years old) populations [5,11]. Whether decreases with aging become significant in the elderly or in earlier ages has not been tested.

The aim of this study was to determine whether the cytokine production in whole-blood-stimulated samples from a young and healthy population is influenced by age, sex, and smoking. In women, the influence of the menstrual cycle was also analyzed.

## 2. Materials and Methods

### 2.1. Study Design and Participants

A descriptive, cross-sectional study was conducted. All participants were informed of the purpose and demands of the study before giving their written consent to participate. The protocol was in accordance with the Declaration of Helsinki for research involving human subjects and was approved by the Balearic Islands Clinical Investigation Ethics Committee (IB 2399/14 PI). All participants were residents in Mallorca (Balearic Islands, Spain). Participants were enrolled after fulfilling all inclusion criteria and presenting none of the exclusion criteria. Participants could be included if they were currently healthy and aged 18–55 years old. Exclusion criteria were common cold, flu or similar diseases, infectious disease or allergic episodes within the previous two weeks, regular alcohol (more than one daily drink in women and two daily drinks in men) or drugs consumption, consumption within the 2 weeks preceding the study of anti-inflammatory medication, and pregnancy. Initially, 255 participants were recruited, but blood could not be obtained from two, and the blood volume taken from three was not enough to perform the stimulation experiences, leading to the final number of 253 participants. Most participants were recruited among students and staff from the University of the Balearic Islands. This allows to ensure that these participants were under similar work-related LPS exposure, one of the factors that could influence cytokine production.

### 2.2. Laboratory Visit

Participants arrived at the laboratory between 08:00 a.m. and 10:00 a.m. following an overnight fast of approximately 12 h. They had been previously informed about the study demands and about the inclusion and exclusion criteria. They had also been instructed to abstain from any moderate-vigorous intensity exercise during the 24 h before arriving at the laboratory. Smokers were asked to refrain from smoking before the visit (since they woke up). Information about the study was reiterated again to the participants, they completed an inclusion/exclusion criteria questionnaire, and they then signed an informed consent form. Each participant was asked to empty their bladder before body mass, height, and body composition were recorded. Stature was measured to the nearest 0.5 cm using a stadiometer (Seca 220 (CM) Telescopic Height Rod for Column Scales, Seca GmbH, Hamburg, Germany). Body mass was measured to the nearest 0.1 kg using an electronic scale (Seca 700, Seca GmbH, Hamburg, Germany). Body mass index (BMI) was calculated as weight (kg) divided by height (m) squared (kg·m^−2^). Smoking habit was ascertained, and participants were classified as smokers or non-smokers. Smokers were asked about the daily number of cigarettes consumed. Female participants were asked about the date of their last menstrual bleed. The period week was ascertained considering this date and a four-week period duration. Participants then sat quietly for 10 min before a blood sample was taken. Seated venous blood samples were collected in suitable Vacutainer® blood collection tubes with ethylenediaminetetraacetic acid (EDTA) or heparin as an anticoagulant (BD, Madrid, Spain). Blood cell numbers (leukocytes, neutrophils, lymphocytes, and monocytes) were quantified in the EDTA blood sample using an automatic flow cytometer analyzer (ABX Pentra 60, Horiba Medical, Montpellier, France). Blood collected with heparin was used for the blood culture experiences.

### 2.3. Blood Culture and LPS Stimulation

Diluted whole heparin blood (1:3 with culture media, RPMI-1640 Medium, Sigma, St Louis, MO, USA) was incubated into sterile 12-well plates (Sarstedt, Nümbrecht, Germany) with lipopolysaccharide (Escherichia coli serotype 055:B5; Sigma, St Louis, MO, USA; final concentration 10 ng/mL), or with the same volume of culture media (spontaneous production) for 24 h at 37 °C. Immediately after incubation, samples were centrifuged at 1000× *g* for 10 min to obtain the supernatants. Aliquots of these culture supernatants were stored at −70 °C until assayed.

### 2.4. Cytokine Measurements

Concentrations of IL-10, IL-6, and TNF-α were determined in the culture supernatants using commercially available enzyme-linked immunosorbent assay kits (Invitrogen, Carlsbad, CA, USA), with a spectrophotometric microplate reader (PowerWavei; BioTek, Winooski, VT, USA). Monocyte numbers were used to normalize cytokine production on a per-cell basis [1,16]. Cytokine-stimulated production is reported as the difference between cytokine concentration in stimulated and unstimulated cultures.

### 2.5. Statistical Analysis

Statistical analysis was carried out using IBM SPSS Statistics 22.0 software (IBM, Chicago, IL, USA). All data were tested for normal distribution (Kolmogorov–Smirnov test), and it was found that cytokine-stimulated production values did not follow a normal distribution. The results were expressed as means and standard deviations (SD), or median and interquartile ranges, as specified. Percentages were also used when required. General characteristics of participants were compared using a *t*-test for unpaired data. The Mann–Whitney U test was used to evaluate differences between sexes and between smoker and non-smoker participants in cytokine-stimulated production. Kruskal–Wallis one-way ANOVA was used to determine the effect of the menstrual cycle week on cytokine production in women, as well as the significance of the changes in cytokine production in participants stratified per age. For this last analysis, five age groups were considered: 18–19, 20–29, 30–39, 40–49, and 50–55 years old. The existence of significant bivariate correlations between cytokine production (logarithmic transformed), age, and BMI was ascertained by determining Pearson correlation coefficients. Multiple linear regression analysis, using the stepwise procedure, was applied to determine the association between each dependent variable (log10 transformed IL-10, IL-6 and TNF-α-stimulated production) and independent (age, sex, and smoking status) and control variables (BMI). Statistical significance was accepted at *p* < 0.05.

## 3. Results

### 3.1. General Characteristics of Participants in the Study

Table 1 shows the general characteristics of participants in the study. Higher values for body mass (*p* < 0.001), stature (*p* < 0.001) and BMI (*p* < 0.001) in men than in women were observed. No differences between men and women were found for age (*p* = 0.534), leukocyte (*p* = 0.681), neutrophil (*p* = 0.363), lymphocyte (*p* = 0.674) and monocyte (*p* = 0.407) counts. Among participants in the study, 32 were smokers (11 men and 21 women), with an average daily consumption of 6.6 ± 10.9 cigarettes.

### 3.2. Culture Cytokine Production

No differences per sex, age, or smoking habit were observed for unstimulated IL-10, IL-6, and TNF-α production (results not shown). Table 2 shows cytokine production in participants stratified by sex. Higher IL-10 (*p* < 0.001) but lower TNF-α (*p* = 0.039) production were observed in women. No differences between sexes were observed for IL-6 production (*p* = 0.068).

Table 3 shows values for cytokine production in non-smoker and smoker participants. Significant lower productions of IL-10 (*p* < 0.001), IL-6 (*p* = 0.003) and TNF-α (*p* = 0.033) were observed in smokers.

**Figure 1:**
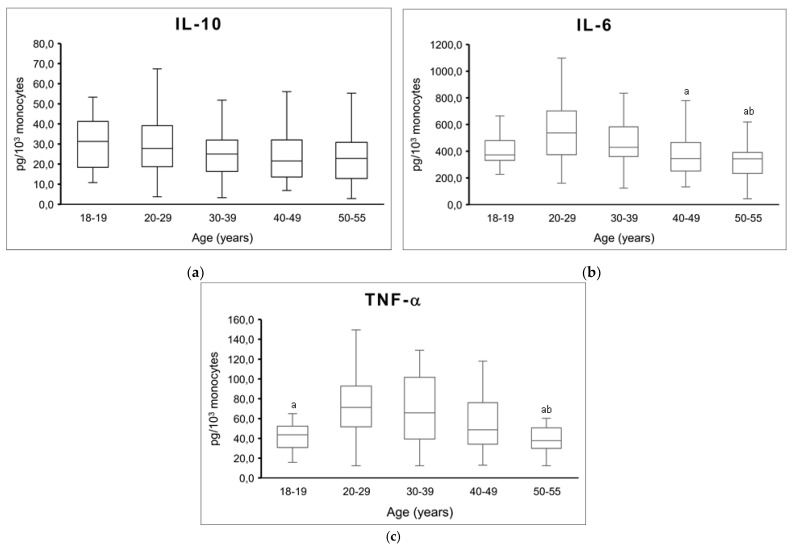
IL-10 (**a**), IL-6 (**b**), and TNF-α (**c**) stimulated production stratified by age groups. Median, 25th, 75th percentile, and lowest and highest values for cytokine production are shown. Age 18–19, *n* = 22; age 20–29, *n* = 82; age 30–39, *n* = 63; age 40–49, *n* = 51; age 50–55, *n* = 35. “a” indicates significant differences vs. 20–29 y group, “b” indicates significant differences vs. 30–39 y group (*p* < 0.05).

The analysis of cytokine production stratified by age groups revealed a significant influence of age on IL-6 (*p* = 0.001) and TNF-α (*p* = 0.003) stimulated production. However, although a decreasing trend in IL-10 production with age was observed, the effect was not significant (*p* = 0.240, Figure 1a). IL-6 production in the group aged 20–29 years was significantly higher than in groups aged 40–49 (*p* = 0.039) and 50–55 (*p* = 0.002) years (Figure 1b). IL-6 production in the group aged 30–39 years was also significantly higher than in the group aged 50–55 years old (*p* = 0.015). TNF-α production in participants aged 20–29 and 30–39 years was higher than in the group aged 50–55 years (*p* = 0.039 and *p* = 0.024, respectively, Figure 1c). Furthermore, TNF-α production was lower in the group aged 18–19 years than in groups aged 20–29 (*p* = 0.039) and 30–39 (*p* = 0.042) years.

### 3.3. Correlations between Cytokine-Stimulated Production, Age, and BMI

When correlations between cytokine production (logarithmic transformations) and continuous dependent variables (age and BMI) were analyzed, it was observed that IL-10 (r = −0.267, *p* = 0.021) and IL-6 (r = −0.267, *p* < 0.001) production were significantly correlated with age. Furthermore, IL-10 production was correlated with BMI (r = −0.159, *p* = 0.012).

### 3.4. Linear Regression Analysis for IL-6-, IL-10-, and TNF-α-Stimulated Production

Table 4 shows the results of the regression analysis for IL-10 production (significant predictors). Model 1 includes smoking as the significant predictor (change in R^2^ = 0.064, *p* < 0.001), with higher IL-10-stimulated production for non-smokers. Model 2 incorporates sex as a significant predictor (change in R^2^ = 0.070, *p* < 0.001), increasing R^2^ adjusted value from 0.064 to 0.134, and with higher IL-10 production for women. Finally, model 3 includes age as a significant negative predictor (change in R^2^ = 0.021, *p* = 0.013), leading to a final R^2^ adjusted value of 0.145, and with IL-10-simulated production decreasing with age (β = −0.145).

Table 5 shows the linear multiple regression models for IL-6- and TNF-α-stimulated production (significant predictors). As it is shown in the previous section, age was not linearly associated with IL-6 and TNF-α production, which supposes one of the assumptions for a variable to be included in the linear regression analysis, mainly due to values observed for participants aged 18 and 19 years old. Therefore, only participants aged 20 and older were included in this linear regression analysis. Among factors considered, age was found to be the main significant predictor (change in R^2^ = 0.089, *p* < 0.001) for IL-6 production (model 1). Within participants considered in this analysis, IL-6-stimulated production decreased with age (β = −0.298). In addition, model 2 includes smoking as a significant predictor (change in R^2^ = 0.037, *p* = 0.002), increasing adjusted R^2^ from 0.085 to 0.118, and with higher productions for non-smokers. In a similar way, model 1 for TNF-α production included age as the significant, and negative, predictor (β = −0.170, change in R^2^ = 0.029, *p* = 0.009). Model 2 for TNF-α production also includes smoking as a significant predictor (change in R^2^ = 0.022, *p* = 0.022), increasing adjusted R^2^ from 0.025 to 0.042. Model 3 for TNF-α-stimulated production considers sex as the last significant predictor (change in R^2^ = 0.016, *p* = 0.045), leading to a final adjusted R^2^ value of 0.067.

### 3.5. Effects of the Menstrual Cycle on the Cytokine-Stimulated Production

Table 6 shows the cytokine production in women stratified per menstrual cycle week. A significant effect of the menstrual cycle on IL-10-stimulated production was observed (*p* = 0.035), with higher production in the second than in the third week (*p* = 0.039). No effects were observed for IL-6 (*p* = 0.681) and TNF-α (*p* = 0.383) production.

## 4. Discussion

The main findings of the present study were that women showed lower production of the pro-inflammatory cytokine TNF-α and higher production of the anti-inflammatory cytokine IL-10 than men. Furthermore, a general decrease in cytokine-stimulated production with aging, as well as lower values in smokers than in non-smokers, were observed.

Sex, age, and smoking have been found as mutually independent determinants of stimulated cytokine production, with a consistent contribution as the regression coefficients of the regression analysis hardly change when another determinant was added to the model (Table 4 and Table 5). Factors considered in the present study (sex, age, and smoking) could explain only a limited fraction of all interindividual variation in cytokine responses. Therefore, additional factors must be considered in future studies to improve the analysis of the cytokine response upon stimulation. However, it should be considered that stimulated cytokine production is under tight genetic control, which could account for over 50% of this variability [4].

Regarding age, a decrease in the production of the cytokines with aging could be considered as the main general observation. In accordance with the decreased stimulated production observed in the present study, others have reported decreased LPS-induced production of TNF-α by isolated monocytes from elderly (79 years old) compared with young (33 years old) humans [5] and also reduced production by stimulated whole blood in participants aged 81 years with respect to participants aged 19–30 years [11]. It is noteworthy that in the present study, decreased production of the pro-inflammatory cytokine TNF-α [5,11], but also of IL-6 [5], was observed for participants aged around 50 years old, while most of the previous studies reported such decreased levels for older ages. Although a previous study [11] did not find a decrease in IL-6-stimulated production, it was pointed out that IL-6 production showed a strong linear correlation with the decreased TNF-α production. The significance of this decreased production is not clear. As it has been previously suggested, the lower production of these cytokines in the elderly may provide an explanation for them becoming more susceptible to infections and having a poor clinical outcome [5]. Furthermore, this decreased production may be of special importance in elderly humans with underlying health disorders, whereas the clinical relevance is questionable in healthy elderly [11]. It has been suggested that ex vivo LPS-stimulated leukocytes TNF-α release could be considered a biomarker of monocytic immune capacity and reflective of global immune competence, the persistent reduction being predictive of adverse outcomes, and its restoration as an indicator of clinical improvement [16,17,18,19].

Regarding changes in stimulated cytokine production with aging, it is noteworthy that TNF-α production was lower in participants aged 18–19 years old than in the ones aged 20–29 years old, a pattern also observed for IL-6 but with non-significant differences. In this regard, as it has been indicated in the Section 3, participants aged 18–19 years were excluded from the regression analysis because when these participants were considered age was not simply linearly associated with cytokine (IL-6 and TNF-α)-stimulated production. However, it should be noted that even after including all participants in the regression analysis, age remained a significant predictor for both IL-6 and TNF-α production (results not shown). However, the models explained a lower percentage of interindividual variation (R^2^ values: IL-6: 0.105 vs. 0.126; TNF-α: 0.032 vs. 0.067). These lower values could be due, at least in part, to the lack of a complete linear fitting between age and stimulated cytokine production described above. Therefore, while we can conclude that aging induced lower cytokine-stimulated production, and it seems that for the pro-inflammatory cytokines the pattern of change in the younger participants is different. The lower pro-inflammatory cytokine production in the youngest participants could be related to incomplete development of the immune system, as it has been suggested that this is completed during the second decade of life [20,21]. In fact, results from the present study suggest that the highest pro-inflammatory cytokine production was reached at 20–29 years old. However, more studies should be performed to further clarify this question.

Sex differences in cytokine-stimulated production were previously reviewed in a study with results from 15 populations [2]. This study concluded that sex differences in cytokine-stimulated production disappear when results were normalized using the monocyte number, except for higher production of IL-10 in women than in men. In agreement with this last observation, a higher stimulated IL-10 production was found in women than in men in the present study (Table 2). Actually, sex was found as a significant predictor for IL-10-stimulated production (Table 4). The significance of this difference is unknown. It has been suggested that an association with reproductive success could exist, as it has been described that reproductive success could be dependent on a strong IL-10 production response at the fetal–maternal interface [22]. However, a previous study in physically active participants reported that a high IL-10-stimulated production was a risk factor for the development of upper respiratory tract infection (URTI) [23]. This effect was attributed to an inhibitory role of IL-10 on Th1 antiviral actions rather than to the inhibition of pro-inflammatory cytokine production [23]. Along the same lines, it has been reported that high IL-10 responders had wheezing symptoms more often than low IL-10 responders [24]. Furthermore, a higher pro-inflammatory cytokine and a lower anti-inflammatory production response upon stimulation with LPS have been associated with survival from meningococcal infection [25], a lower incidence of systemic lupus erythematosus [26], and less severe progression of multiple sclerosis [27]. Within the healthy participants in the present study, in addition to higher IL-10 production, lower TNF-α production in women was observed, which is in agreement with previous observations [24,28]. This decreased value in women has, in general, been attributed to the influence of sex hormones, mainly estrogens [28]. The lower TNF-α production in women than in men could be one of the factors causing the lower incidence of sepsis among women [29].

Chronic smoking has been shown to induce an important impairment in the host defense, with nicotine playing an important role [30]. However, it is striking that a potential therapeutic anti-inflammatory role has been attributed to nicotine, preventing some inflammatory and neurodegenerative diseases [30]. Regarding cytokine-stimulated production, it has been reported that the suppressive effect of smoking is restricted to the lungs [31]. In the present study, decreased stimulated productions of the three cytokines tested were found. The decreased IL-10 production could be attributed to nicotine because, in different cellular models, a suppressive effect of nicotine exposure on the IL-10-stimulated production has been found [32,33]. It is noteworthy that, in the present study, lower monocyte numbers were found in smokers (results not shown), which could have influenced the results observed. The decrease in IL-6 and TNF-α production could be in agreement with previous results reporting decreased pro-inflammatory production in stimulated alveolar macrophages [31]. However, complete molecular mechanisms leading to this impaired immune function in smokers remain to be fully elucidated.

Previous studies have reported that monocytes produce lower levels of pro-inflammatory cytokines both after LPS stimulation [34] and spontaneously [35] in the presence of high concentrations of female hormones. In the present study, within female participants, differences were found in the stimulated production of IL-10 during the menstrual cycle, with higher production in the second than in the third week (Table 6). These results could be explained by changes in the concentration of estrogen during the menstrual cycle. In this regard, while the third week is characterized by low concentrations of estrogen, with a pro-inflammatory effect, the second week is characterized by higher levels of estrogen, with potent anti-inflammatory effects [36,37]. In fact, despite being non-significant, slightly increased production of IL-6 and TNF-α were observed in the present study during the third week, which could be a consequence of the pro-inflammatory characteristics of this week. However, it is noteworthy that a previous study, using a different approach, through which 12 cycles in only 5 women were analyzed, did not find any change in the cytokine-stimulated production during the menstrual cycle [38].

This study presents some limitations that should be acknowledged. The production of only two pro-inflammatory and one anti-inflammatory cytokine was measured. This, together with the observational nature of the study, could hamper, at least in part, a conclusive and comprehensive interpretation of the effects of different factors analyzed. Furthermore, participants in the study were recruited only in Mallorca, and therefore, the results of the study could not be generalized elsewhere. The low number of smokers considered, together with the lack of additional measurements related to mechanisms involved, such as nicotine or cotinine levels, did not allow proper conclusions to be drawn regarding the effect of smoking. Stimulation for 24 h was chosen to optimize the production of IL-10, as it has been reported that stimulated IL-10 production is secondary to the release of proinflammatory cytokines [39]. However, this may have resulted in missing differences in IL-6 and TNF-α production, which are typically induced earlier, increasing immediately after incubation and peaking 4 h after [39]. Furthermore, it should also be considered that while other studies used isolated monocytes, in this study, LPS stimulation of whole-blood cultures was used. It has been considered that this model probably comes closest to the natural environment, avoiding artifacts from preparation and allowing natural interactions [3]. Regarding the analysis of age influence, it should be noted that age groups considered in the analysis of cytokine production were arbitrarily chosen, and this distribution resulted in different numbers of participants in each age group, with a higher number of participants aged 20–29 years. Hormone levels were not measured in women to find out the effects of hormone concentrations on cytokine production. Therefore, an objective association between hormone levels and cytokine production could not be established.

## 5. Conclusions

Sex, age, and smoking were found to be independent determinants of stimulated cytokine production. While the female sex was associated with higher IL-10 and lower TNF-α production, aging was associated with lower levels of stimulated IL-6, TNF-α, and IL-10 production. Regarding changes with age, it is noteworthy that significantly decreased values for IL-6 and TNF-α production were observed in individuals aged around 50 years old. Therefore, when LPS-stimulated cytokine production is analyzed in patients with diseases, the effect of age, and also differences between sexes, should be considered as confounding factors.

## Figures and Tables

**Table 1:** General characteristics of participants in the study.

Parameter	All (*n* = 253)	Men (*n* = 120)	Women (*n* = 133)	*p* Value
Age (years)	33.0 ± 10.9	33.4 ± 11.0	32.6 ± 10.9	0.534
Body mass (kg)	67.0 ± 13.5	76.3 ± 11.8	58.6 ± 8.6	<0.001 *
Stature (cm)	170.0 ± 9.6	177.0 ± 7.1	163.6 ± 6.4	<0.001 *
BMI (kg·m^−2^)	23.1 ± 3.4	24.3 ± 3.3	22.0 ± 3.2	<0.001 *
Leukocytes (10^3^·μL^−1^)	5.84 ± 1.38	5.80 ± 1.18	5.87 ± 1.55	0.681
Neutrophils (10^3^·μL^−1^)	3.13 ± 0.98	3.07 ± 0.84	3.18 ± 1.08	0.363
Lymphocytes (10^3^·μL^−1^)	1.98 ± 0.60	1.96 ± 0.59	1.99 ± 0.60	0.674
Monocytes (10^3^·μL^−1^)	0.49 ± 0.41	0.52 ± 0.37	0.47 ± 0.44	0.407

Values are expressed as means ± S.D. * indicates significant differences between men and women (*p* < 0.05).

**Table 2:** Cytokine-stimulated production in participants stratified by sex.

Cytokine	Men (*n* = 120)	Women (*n* = 133)	*p* Value
IL-10 (pg·10^−3^ cells)	20.6 (13.5, 28.4)	29.1 (17.4, 42.1)	<0.001 *
IL-6 (pg·10^−3^ cells)	372.8 (262.7, 536.6)	428.8 (331.7, 586.4)	0.068
TNF-α (pg·10^−3^ cells)	56.0 (33.9, 85.3)	49.4 (30.8, 69.1)	0.039 *

Values are expressed as median (25th, 75th percentile) and represent the difference between cytokine concentration in stimulated and unstimulated cultures. * indicates significant differences between sexes (*p* < 0.05).

**Table 3:** Cytokine-stimulated production in smoker and non-smoker participants.

Cytokine	Non-smokers (*n* = 221)	Smokers (*n* = 32)	*p* Value
IL-10 (pg·10^−3^ cells)	25.0 (16.3, 35.7)	14.3 (10.5, 22.6)	<0.001 *
IL-6 (pg·10^−3^ cells)	397.8 (299.0, 562.0)	288.3 (223.2, 445.4)	0.003 *
TNF-α (pg·10^−3^ cells)	53.0 (34.1, 81.5)	42.1 (26.8, 60.0)	0.033 *

Values are expressed as median (25th; 75th percentile) and represent the difference between cytokine concentration in stimulated and unstimulated cultures. * indicates significant differences between smokers and non-smokers (*p* < 0.05).

**Table 4:** Regression models for IL-10-stimulated production.

	B	β	95%CI	t	*p* Value	R^2^	Adjusted R^2^	*p* Value (ANOVA)	R^2^ Change
***Model 1***						0.064	0.060	<0.001 *	0.064
Smoking	−0.196	−0.253	(−0.289, −0.103)	−4.148	<0.001 *				
***Model 2***						0.134	0.127	<0.001 *	0.070
Smoking	−0.216	−0.280	(−0.306, −0.126)	−4.731	<0.001 *				
Sex	0.137	0.266	(0.077, 0.197)	4.504	<0.001 *				
***Model 3***						0.155	0.145	<0.001 *	0.021
Smoking	−0.220	−0.285	(−0.309, −0.131)	−4.859	<0.001 *				
Sex	0.134	0.261	(0.075, 0.194)	4.459	<0.001 *				
Age	−0.003	−0.145	(−0.006, −0.001)	−2.490	0.013 *				

Logarithmic transformation of IL-10-stimulated production was used. *n* = 253. B: regression coefficient; β: standardized beta coefficient; * indicates statistically significant predictors and models (*p* < 0.05). The negative coefficient for smoking indicates lower values for IL-10-stimulated production in smokers than in non-smokers. The positive coefficient for sex indicates higher values in women than in men.

**Table 5:** Regression models for IL-6- and TNF-α-stimulated production.

	B	β	95%CI	t	*p* Value	R^2^	Adjusted R^2^	*p* Value (ANOVA)	R^2^ Change
**IL-6**									
***Model 1***						0.089	0.085	<0.001 *	0.089
Age	−0.006	−0.298	(−0.009, −0.004)	−4.744	<0.001 *				
***Model 2***						0.126	0.118	<0.001 *	0.037
Age	−0.006	−0.311	(−0.009, −0.004)	−5.032	<0.001 *				
Smoking	−0.123	−0.193	(−0.201, −0.045)	−3.119	0.002 *				
**TNF-α**									
***Model 1***						0.029	0.025	0.009 *	0.029
Age	−0.005	−0.170	(−0.009, −0.001)	−2.623	0.009 *				
***Model 2***						0.051	0.042	0.002 *	0.022
Age	−0.005	−0.180	(−0.009, −0.002)	−2.796	0.006 *				
Smoking	−0.137	−0.148	(−0.254, −0.020)	−2.309	0.022 *				
***Model 3***						0.067	0.055	0.001 *	0.016
Age	−0.005	−0.181	(−0.009, −0.002)	−2.836	0.005 *				
Smoking	−0.137	−0.132	(−0.239, −0.005)	−2.309	0.041 *				
Sex	−0.081	−0.129	(−0.160, −0.002)	−3.063	0.045 *				

Logarithmic transformation of IL-6- and TNF-α-stimulated production was used. *n* = 231. B: regression coefficient; β: standardized beta coefficient; * indicates statistically significant predictors and models (*p* < 0.05). The negative coefficient for smoking indicates lower stimulated production in smokers than in non-smokers. The negative coefficient for sex indicates lower values in women than in men.

**Table 6:** Cytokine-stimulated production Cytokine production in women stratified per menstrual cycle week.

Cytokine	Week 1 (*n* = 37)	Week 2 (*n* = 31)	Week 3 (*n* = 28)	Week 4 (*n* = 37)	*p* Value
IL-10 (pg·10^−3^ cells)	31.3 (20.8; 44.6)	42.3 (29.2; 51.4) a	25.9 (19.0; 38.1)	31.3 (14.7; 41.4)	0.035 *
IL-6 (pg·10^−3^ cells)	462.8 (346.3; 610.6)	414.7 (349.1; 569.5)	470.8 (354.9; 709.4)	448.8 (342.1; 618.9)	0.681
TNF-α (pg·10^−3^ cells)	52.1 (29.3; 85.7)	43.4 (31.8; 58.4)	57.7 (39.6; 80.7)	51.3 (32.3; 78.7)	0.383

Values are expressed as median (25th; 75th percentile). *n* = 133. *p*-adjusted values are reported. * indicates statistically significant differences where “a” indicates significant differences between the third and the second week (*p* < 0.05).

## Data Availability

The data presented in this study are available on request from the corresponding author.
